# Biomechanical characterization of a novel ring connector for sutureless aortic anastomosis

**DOI:** 10.7555/JBR.31.20170011

**Published:** 2018-05-28

**Authors:** Huan Liu, Shi-jiang Zhang, Yong-feng Shao, Xiao-hu Lu, Wei-dong Gu, Buq-ing Ni, Qun Gu, Jun-jie Du

**Affiliations:** Department of Cardiovascular Surgery, The First Affiliated Hospital, Nanjing Medical University, Nanjing, Jiangsu 210029, China.; Department of Cardiovascular Surgery, The First Affiliated Hospital, Nanjing Medical University, Nanjing, Jiangsu 210029, China.; Department of Cardiovascular Surgery, The First Affiliated Hospital, Nanjing Medical University, Nanjing, Jiangsu 210029, China.; Department of Cardiovascular Surgery, The First Affiliated Hospital, Nanjing Medical University, Nanjing, Jiangsu 210029, China.; Department of Cardiovascular Surgery, The First Affiliated Hospital, Nanjing Medical University, Nanjing, Jiangsu 210029, China.; Department of Cardiovascular Surgery, The First Affiliated Hospital, Nanjing Medical University, Nanjing, Jiangsu 210029, China.; Department of Cardiovascular Surgery, The First Affiliated Hospital, Nanjing Medical University, Nanjing, Jiangsu 210029, China.; Department of Cardiovascular Surgery, The First Affiliated Hospital, Nanjing Medical University, Nanjing, Jiangsu 210029, China.

**Keywords:** aortic diseases, sutureless device, aortic anastomosis, swine

## Abstract

The surgical treatment for aortic diseases remains a challenge for any cardiac surgeon. The use of sutureless ring connector in aortic anastomosis can simplify the procedure and shorten anastomosis time. Therefore, we developed a novel device for sutureless aortic anastomosis. A series of experiments were carried out for tensile and leakproof-capacity assessments to verify the feasibility of the ring connector by using fresh swine aorta samples. In *in vivo* test, the ring connector was implanted in 6 swine with follow-up of 6 months. Radiographic and pathological studies of the aorta were performed. In the tensile tests, the strength was 32.7±5.9 Newton (N) in the sutureless anastomosis group, compared with 73.3±12.5 N in the control group by traditional manual suture. In the leakproof-capacity assessment, no sign of either leakage or bursting was evident at 280 mmHg of internal pressure in the aorta samples. In *in vivo* tests, it took 9.47±0.3 minutes for the sutureless anastomosis, compared with 15.58±1.39 minutes for hand-sewn suturing. Insertion was easy and rapid. Radiographic and pathological studies were performed at first month, third month and sixth month after surgery, each time obtained from the two swine, showed patency of the anastomosis and no signs of stenosis, blood leakage, migration or pseudoaneurysm formation, except one paralyzed swine developed of thrombo-occlusion at the site of the sutureless anastomosis. The result indicates that this novel ring connector offers considerable promise for sutureless aortic anastomosis.

## Introduction

Since the beginning of cardiovascular surgery, anastomoses have been done with hand-sewn sutures based on the principles of the suture technique described by Alexis Carrel^[1]^. While still the gold standard, in many cases conventional hand-sewn suturing needs dexterity of surgery techniques and technology accumulation, and is also time-consuming, especially in great artery surgery. The surgical treatment for aortic diseases such as aortic aneurysm, aortic dissection and aortic trauma remains a challenge for any cardiac surgeon. Although the use of bypass, deep hypothermia circulation arrest technique or extracorporeal circulation may reduce procedure-related complication rates, the key is to improve the speed of aortic repair. Improvements in surgical techniques that reduce clamp time and make the procedure technically easier would be of benefit. In recent decades, for the sake of reducing aortic anastomosis operation time, new technologies have surged such as sutureless device for aortic anastomosis^[2^–^7]^. Use of the sutureless device reduces clamp time and can minimize the incidence of time-related complications. Another advantage of the sutureless device is avoidance of difficult sutured anastomosis in patients with a severely diseased aorta. Therefore, we have developed a novel sutureless anastomosis device for end-to-end anastomosis between the aorta and an artificial vessel. The purpose of this study was to assess the function and safety of the sutureless anastomosis device, and to test it *in vitro*, and *in vivo*.

## Materials and methods

### Description of the ring connector

**Fig.1:**
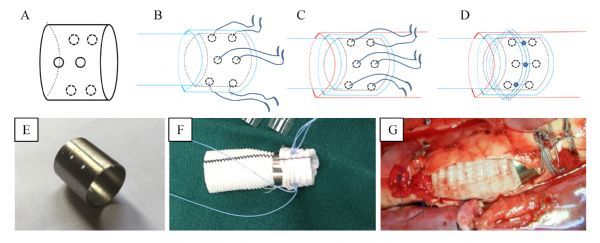
Features of the design and use of the ring connector.

The ring connector (20 mm in length) is composed of biocompatible and biological nontoxic titanium alloy (Ti-6Al-4V) and has various diameters. There are three pairs of holes on the surface, which are located in the different circumferential orientation, each forming an angle of 120 degrees. When used, the vessel prosthesis traverses the ring from inside and the end is rolled over the ring. The prosthesis and the ring are fixed with double-armed 2-0 polypropylene sutures through the three pairs of holes. When anastomosis is performed, the ring connector covered with vessel prosthesis is inserted into the aorta and each of the double-armed 2-0 polypropylene sutures traverses the aortic wall and is fixed with a tie around the outer surface of the aorta. The three ligatures of stitches were performed to guarantee the reliability of anastomosis (***Fig. 1***).

### ***In vitro*** testing

A series of experiments were carried out to verify the feasibility of the ring connector by using fresh swine aorta samples. The tensile and leakproof-capacity assessments were tested by bench experiments. The dissected arteries (*n* = 8) were anastomosed with the ring connector onto woven vascular prosthesis (Gelweave^TM^); for control tests (*n* = 8), the anastomosis were finished by conventional manual suture. In tensile tests, we intended to measure the resistance of the anastomosis connected by using our device. The tensile strength of the test and control anastomosis, quantified in Newtons (N), was measured by dynamometer. The tensile stress was increased until the anastomosis break point, and the measured tensile force was recorded. In leakproof-capacity test, each end of the anastomosis was sealed and a compressive infusion set was applied to simulate arterial pressure up to 280 mmHg by injecting saline into the vessel.

### ***In vivo ***animal studies

#### Animals

Six adult Chinese indigenous pigs (3 males) weighing about 80 kg were used in this study. All animal testing procedures were approved by our Institution's research and ethic committee which adheres to the International Guiding Principles for Animal Research. All surgeries were performed in designated veterinary operating room with sterilized instruments and procedure.

#### Surgical procedure

Anesthesia was induced and maintained with diazepam (0.6 mg/kg), proazamine (1.2 mg/kg), atropine sulfate (0.03 mg/kg) and ketamine (14 mg/kg) intravenously after intubation and ventilation. The abdominal aorta of each swine was exposed through a thoracotomy in the left fifth intercostal space. When the descending aorta was free, arterial bypass was established across the target anastomotic location by using an annular tube after intravenous administration of heparin with a dosage of 1.5 mg/kg. An aortotomy was made at the site of anastomotic location and the ring connector attached to artificial vascular graft was inserted into the artery. The proximal end of the anastomosis was completed in a sutureless way with our novel ring connector while the distal end was finished by traditional suture technique as control. The times required for both the proximal and distal anastomoses were recorded. The abdominal incision was then closed.

#### Postoperative management

Intramuscular injection of penicillin 800,000 units was used daily at the first week after the operation and all swine were fed a normal diet without any anticoagulant Drugs added.

#### CT angiography

At the first, third and sixth month after the surgery, contrast-enhanced CT angiography (CTA) was performed to visualize the entire thoracic aortic vasculature on two of the swine each time.

#### Pathological analysis

After the animals were sacrificed, the descending aorta specimens were collected including both sides of the anastomoses for pathological assessment.

### Statistical analysis

Continuous variables were expressed as mean±standard deviation. SPSS version 19.0 (SPSS Inc., Chicago, IL, USA) was used for all analyses. An independent-sample *t*-test was performed to check for differences between the two kinds of anastomosis. The level of significance was set at *P* 0.05.

## Results

### ***In vitro*** testing

**Fig.2:**
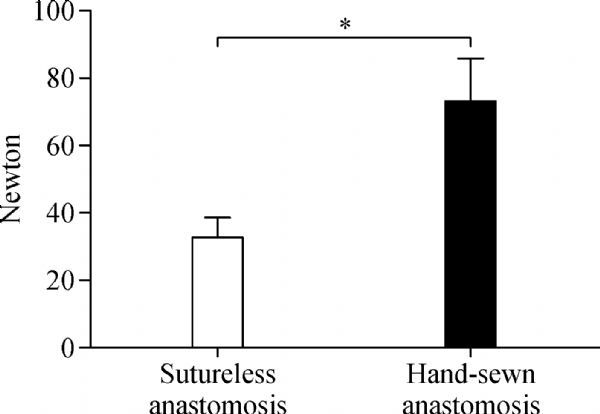
The tensile strength of the sutureless and hand-sewn anastomosis when performed on fresh swine aorta samples (*n* = 8, *P 0.001)

In the tensile tests using fresh swine aorta samples, the strength was 32.7±5.9 Newton (N) in the sutureless anastomosis group, compared with 73.3±12.5 N in the control group by conventional manual suture (***Fig. 2***). The results showed that hand-sewn anastomoses were significantly stronger than sutureless anastomoses. In the leakproof-capacity assessment, no sign of either leakage or bursting was evident at 280 mmHg of internal pressure in the aorta samples.

### ***In vivo ***animal studies

#### Surgery

**Fig.3:**
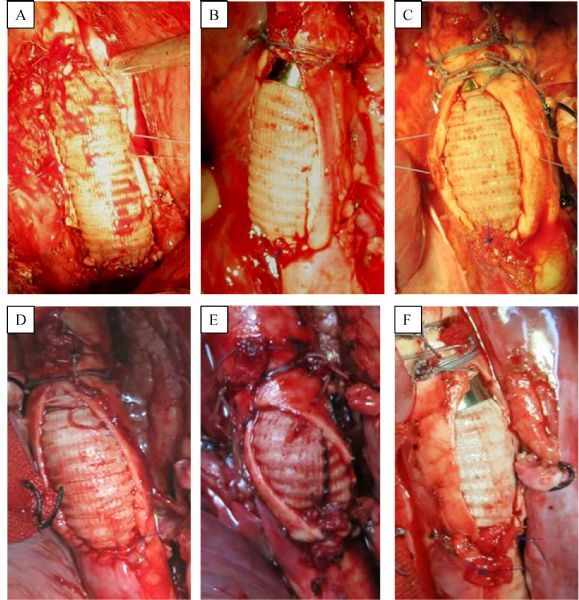
View after completion of the anastomoses on six swine during the surgeries.

**Fig.4:**
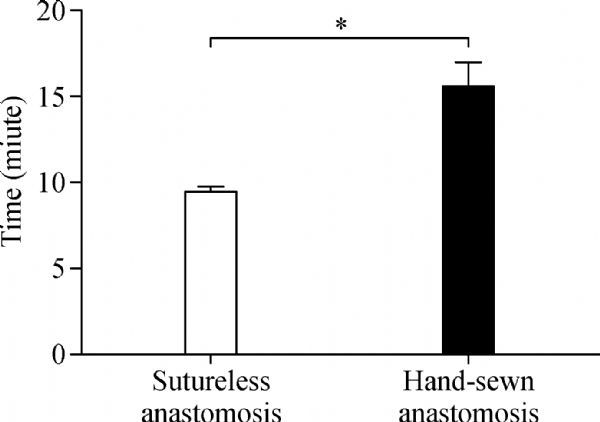
Time required for sutureless and hand-sewn anastomosis (***n*** = 6, *P = 0.001).

The operative procedure was completed in all six swine without any complications except that one progressed to rear limb paralysis (***Fig. 3***). The sutureless anastomotic procedure was completed within 10 min in all six cases. It took 9.47±0.3 minutes for sutureless anastomosis, compared with 15.58±1.39 minutes for hand-sewn suturing. The time required for device assisted sutureless anastomosis was significantly less than for manual suture anastomosis (***Fig. 4***). All animals were alive at the scheduled time point. Thrombo-occlusion was observed only in one animal, which was development of rear limb paralysis after the surgery, although no other problems such as blood leakage, migration and pseudoaneurysm formation were found at both sutureless and manual suture anastomoses.

#### CT angiography

**Fig.5:**
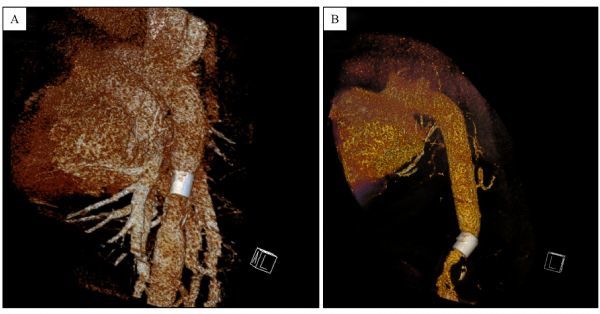
View of three-dimensional reconstruction of the aorta.

CT angiography at the first, third and sixth month after surgery, each time obtained from two swine, showed patency of the anastomosis and no signs of stenosis, blood leakage, migration or pseudoaneurysm formation (***Fig. 5***), except that the paralyzed one developed of thrombo-occlusion at the site of sutureless anastomosis.

#### Pathological analysis

**Fig.6:**
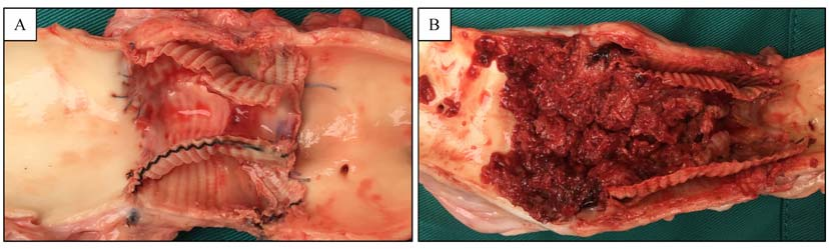
Macroscopic view of a longitudinal section of the aorta. The device had been removed.

Macroscopic examination confirmed intimal healing of the aorta to the vascular graft, the specimen of the paralyzed swine showed thrombus formation while others exhibited no evident thrombus and fibrin deposition at the site of anastomosis (***Fig. 6***).

## Discussion

These *in vitro* testing results demonstrated that the new sutureless device constructed an anastomosis with reliable mechanical strength and leakproofcapacity. Although hand-sewn anastomoses were significantly stronger than sutureless anastomoses, the strength of sutureless anastomoses was strong enough to guarantee the integrity and reliablity of anastomosis when performed on the aorta. In animal study, using this newly developed ring connector device, the anastomosis between the thoracic descending aorta and vessel prosthesis can be finished within a relative short time period.

The surgical treatment for aortic diseases remains a challenge for cardiothoracic surgeons. Hand sewing aortic anastomosis of the vessel prosthesis and the endogenous vessels is very time consuming, and inevitable bleeding during surgery is sometimes formidable to control, and the fragility of atherosclerotic aortic walls also makes suturing difficult. Paraplegia and brain damage are devastating complications after aortic reconstruction while the prolonged pumping time is one of the major risks in spite of some methods for their prevention such as deep hypothermic arrest, antegrade and retrograde cerebral perfusion^[8^–^10]^. Hand-sewn vascular anastomoses have potential disadvantages including lasting anastomotic time, high technical requirement, and suture-hole bleeding; therefore, the use of sutureless anastomotic devices that can decrease surgery time and simplify the procedure ought to be considered. After decades of development, there is still a huge controversy whether sutureless device should be applied for aortic anastomosis, which has really hampered its clinical use. Potential problems are still of concern including anastomotic leaks, migration, thromboembolism, and pseudoaneurysm formation from erosion or tears due to shear forces related to the rigid ring, or from pressure necrosis produced by fixation ligatures^[11^–^13]^.

Sutureless aortic anastomosis device was always designed with a rigid ring connector and vessel prosthesis. The surface of the ring is smooth, and a shallow groove is designed for fixed vessels by tapes; however, it is still possible that the aorta slips from the connector which leads to fatal consequences. The groove limits the vessel lumen diameter and blood flow that may lead to the occurrence of thrombosis or other complications. The tightened aortic tissue around the vascular ring connector could have become weak and thus would be subject to late disruption, although the reported pathological findings alleviated concerns about this potential problem^[14]^. Some expandable prostheses make themselves more convenient to be inserted into the aorta, but the procedure and use of the device is more complex, and the sophisticated design also increases the manufacturing cost^[15^–^17]^. Some researchers tried a luminal stent with spikes, which may bring about a huge vascular damage, and the procedure is not reversible^[7]^. Once operational errors occur, the damaged blood vessels can only be removed. The use of new materials, such as biodegradable stents, shape memory alloy, as well as some other sutureless anastomosis means such as staples and tissue adhesives still need more experimental and clinical studies^[18^–^21]^.

The main advantage of our ring connector device is its simplicity, which shortens the time required for aorta and vascular prosthesis anastomosis while maintaining satisfactory quality. With the help of this device, the vessel clamping time was significantly reduced, which may prevent the occurrence of related complications. The second merit of this technique is that titanium alloy ring connector had no direct contact with blood that prevents the occurrence of some possible adverse effects. What's more, our device fixed the vascular prosthesis and aorta by transfixion through three pairs of holes, unlike the other designs with groove, will not limit the vessel lumen diameter and blood flow.

Thrombo-occlusion was observed at the sutureless anastomosis only in one animal who developed rear limb paralysis after the surgery. Several possible reasons may have contributed to this phenomenon. First, the paralyzed swine was the first experimental subject when our operative process was not mature enough, that may result in some unpredictable injury to the aorta. Secondly, no swine received anticoagulation therapy after the surgery, just like the treatment we adopted on human being. However swine exert a tendency to hypercoagulability which may lead to thrombus formation without anticoagulant therapy^[22]^. Thirdly, for this preliminary experimental study, the ring connector and the prosthesis were separated and when it was used, the vessel prosthesis traversed the ring from inside and the end was rolled over the ring. In that way, the prosthesis may not be attached to the ring connector's inside wall well and formed several folds. Hemodynamic could be disturbed when blood flows through these folds to facilitate the formation of thrombus^[23^–^24]^. Future designs will be finished in the production of artificial blood vessels directly bonded with the inner wall of the ring connector to avoid possible induction of thrombosis or stenosis.

In this study, we showed that anastomosis with the assistance of our novel device exhibited significantly fewer complications and was less time-consuming than the conventional technique. Due to the use of a few large animals, the proximal anastomosis was performed with the ring connector and distal anastomosis was hand-sewn. This study was merely intended as a first step to test the feasibility of our novel device. Further works are necessary to assess the function and practicability of this ring connector with long-term follow-up.

In conclusion, the use of our novel ring connector in aortic anastomosis can simplify the procedure and shorten anastomosis time. The device also has proven to be feasible and effective during a 6-month observation on large animals. The device might be a good alternative to common suture technique, while its usefulness in the routine treatment of aortic diseases warrants further evaluation.
